# Preconception nutrition intervention improved birth length and reduced stunting and wasting in newborns in South Asia: The Women First Randomized Controlled Trial

**DOI:** 10.1371/journal.pone.0218960

**Published:** 2020-01-29

**Authors:** Sangappa M. Dhaded, K. Michael Hambidge, Sumera Aziz Ali, Manjunath Somannavar, Sarah Saleem, Omrana Pasha, Umber Khan, Veena Herekar, Sunil Vernekar, Yogesh Kumar S., Jamie E. Westcott, Vanessa R. Thorsten, Amaanti Sridhar, Abhik Das, Elizabeth McClure, Richard J. Derman, Robert L. Goldenberg, Marion Koso-Thomas, Shivaprasad S. Goudar, Nancy F. Krebs

**Affiliations:** 1 Women’s and Children’s Health Research Unit, KLE Academy of Higher Education and Research, Jawaharlal Nehru Medical College, Belagavi, Karnataka, India; 2 Pediatric Nutrition, University of Colorado School of Medicine, Aurora, Colorado, United States of America; 3 Aga Khan University, Karachi, Pakistan; 4 RTI International, Durham, North Carolina, United States of America; 5 Thomas Jefferson University, Philadelphia, Pennsylvania, United States of America; 6 Department of Obstetrics/Gynecology, Columbia University Medical Center, New York, New York, United States of America; 7 NICHD/NIH, Rockville, Maryland, United States of America; Institut de recherche pour le developpement, FRANCE

## Abstract

South Asia has >50% of the global burden of low birth weight (LBW). The objective was to determine the extent to which maternal nutrition interventions commenced before conception or in the 1^st^ trimester improved fetal growth in this region. This was a secondary analysis of combined newborn anthropometric data for the South Asian sites (India and Pakistan) in the Women First Preconception Maternal Nutrition Trial. Participants were 972 newborn of mothers who were poor, rural, unselected on basis of nutritional status, and had been randomized to receive a daily lipid-based micronutrient supplement commencing ≥3 months prior to conception (Arm 1), in the 1^st^ trimester (Arm 2), or not at all (Arm 3). An additional protein-energy supplement was provided if BMI <20 kg/m^2^ or gestational weight gain was less than guidelines. Gestational age was established in the 1^st^ trimester and newborn anthropometry obtained <48-hours post-delivery. Mean differences at birth between Arm 1 vs. 3 were length +5.3mm and weight +89g. Effect sizes (ES) and relative risks (RR) with 95% CI for Arm 1 vs. 3 were: length-for-age Z-score (LAZ) +0.29 (0.11–0.46, p = 0.0011); weight-for-age Z-score (WAZ) +0.22 (0.07–0.37, p = 0.0043); weight-to-length-ratio-for-age Z-score (WLRAZ) +0.27 (0.06–0.48, p = 0.0133); LAZ<-2, 0.56 (0.38–0.82, p = 0.0032); WAZ <-2, 0.68 (0.53–0.88, p = 0.0028); WLRAZ <-2, 0.76 (0.64–0.89, p = 0.0011); small-for-gestational-age (SGA), 0.74 (0.66–0.83, p<0.0001); low birth weight 0.81 (0.66–1.00, p = 0.0461). For Arm 2 vs. 3, LAZ, 0.21 (0.04–0.38); WAZ <-2, 0.70 (0.53–0.92); and SGA, 0.88 (0.79–0.97) were only marginally different. ES or RR did not differ for preterm birth for either Arm 1 vs. 3 or 2 vs. 3. In conclusion, point estimates for both continuous and binary anthropometric outcomes were consistently more favorable when maternal nutrition supplements were commenced ≥3 months prior to conception indicating benefits to fetal growth of improving women’s nutrition in this population.

## Introduction

South Asian (S. Asian) countries, including India, Pakistan, Nepal, and Bangladesh, have wide intra- and inter-cultural diversity but much in common to encourage a regional perspective on women’s and children’s nutrition [1]. Despite recent declines in frequency of stunting, 38% of children under 5 years of age in S. Asia are still stunted, with rates exceeding one-third each in Pakistan, India, Bangladesh, and Nepal [2]. This region, because of the size of the population and high rates of stunting, accounts for 40% of the global burden of stunting [2–7]. Though wasting rates for children under the age of 5 years are lower, they remain unacceptably high, especially in India, as do rates of underweight in women of childbearing age. Only in Sri Lanka and Maldives is undernutrition now over-shadowed by excessive rates of obesity and diabetes [2]. The causes of early childhood stunting in S. Asia, as elsewhere, are multifactorial. However, low birth weight, especially low birth size for gestational age is a major factor [3, 8–10]. S. Asia accounts for over 50% of the global occurrence of low birth weight (LBW), with both Pakistan and India being major contributors to this high figure [11]. Furthermore, it has been postulated quite convincingly that recent figures are underestimates [9]. Even relatively mild fetal growth retardation is a strong predictor of stunting by two years of postnatal age. This applies whether the incidence of fetal growth failure is evaluated with birth weight or/and length measurements [12].

The importance of fetal growth directs attention to the in utero and, therefore, the maternal environment. Poverty is a common and overriding environmental cause of population fetal and postnatal growth retardation, and the apparent ‘enigma’ of the very high level of stunting and wasting in S. Asia has also been attributed specifically to the low social status of women in poor rural communities [13]. However, other factors contribute, among which malnutrition has a prominent primary and secondary role requiring specific preventive measures. In one study, birth size was strongly positively correlated with the intake of micronutrient-rich foods and with milk and fat intake [14]. Recent reviews of childhood stunting in S. Asia have included recognition of the importance of maternal nutrition and have given attention to the nutrition of non-pregnant women [4, 10, 15–20]. Priority attention to eliminating maternal undernutrition and fetal growth retardation are recognized to be central to health and sustainable development in S. Asia and elsewhere [21–24]. Public health measures focused on attention to nutrition at a community level are contributing to recent progress in India, Nepal, Bangladesh, and elsewhere [25–29]. However, current statistics reinforce the need to both ramp-up the application of existing evidence-based strategies and to test new strategies to improve women’s and, therefore, fetal nutrition [13].

Here we report the newborn anthropometric data for the combined S. Asian sites participating in the Women First Preconception Maternal Nutrition Trial [30].

## Objectives

The primary objective of this study was to determine the quantitative improvements in the deficits in birth anthropometry resulting from commencing maternal nutrition supplements at least three months prior to conception or in the first trimester of pregnancy in the combined S. Asian sites participating in the Women First Preconception Maternal Nutrition Trial.

## Methods

### Ethics

The project was approved by the Colorado Multiple Institutional Review Board, University of Colorado; JNMC Institutional Ethics Committee on Human Subjects Research and the Indian Council of Medical Research (India); and The Aga Khan University Ethical Review Committee (Pakistan); and the Data Coordinating Center. All of these are registered with US Office of Human Research Protection and with Federal-wide Assurance in place. Written informed consent was obtained from all participants. The study protocol is available online: https://www.ncbi.nlm.nih.gov/pmc/articles/PMC4000057/. The Clinical Trial Registry number and website is ClinicalTrials.gov #NCT01883193; https://clinicaltrials.gov/ct2/show/NCT01883193?term=01883193&rank=1.

### Study design and outcomes

This study was a secondary analysis of neonatal anthropometric outcomes in the Women First trial [30]. The parent study was an individually randomized, non-masked, multi-site randomized controlled efficacy trial conducted in four sites. The study sites are part of the of the *Eunice Kennedy Shriver* NICHD Global Network for Women’s and Children’s Health Research (GN) and are composed of a total of 24 geographic clusters from the GN Maternal Newborn Health Registry Each cluster has approximately 300 deliveries per year [31]. The trial included three arms: Arm 1 started the nutrition supplement for pregnant women at least three months prior to conception; Arm 2 commenced the same supplement near the end of the first trimester; and Arm 3 which did not receive any trial supplement. This South Asian sub-study was not included in the original Women First Preconception trial protocol or data analysis plan but was conceived as a separate sub-study prior to data analyses for those maternal / newborn dyads who had gestational age determined by ultrasound in the first trimester.

The continuous outcomes reported here by study arm include newborn length, weight, head circumference (HC), length-for-age Z-score (LAZ), weight-for-age Z-score (WAZ), HC-for-age Z-score (HCAZ), and weight to length ratio-for-age Z-scores (WLRAZ). Categorical outcomes include small for gestational age (SGA, weight <10^th^ percentile for gestational age), low birth weight (LBW, <2500 g), preterm birth (PTB, gestational age <37 weeks), LAZ <-2, WAZ <-2, WLZ <-2 and WLRAZ <-2. Also included are RR for Z-scores <-1 because of their predictive value for subsequent infant growth [12].

### Subjects and randomization

The participants included in this study were newborns whose mothers met all criteria for inclusion and retention in the parent study and who had ultrasound measurements of crown-rump length (CRL) in the first trimester. They were located in resource-poor rural communities in two S. Asian countries (India and Pakistan) and enrolled into the study between January and December 2014; data collection was completed in March 2017. The mothers of newborns in Arms 1 and 2 did not receive non-trial nutrition supplements during the trial intervention period. A high percentage of Arm 3 mothers in the India site received iron-folate supplements commencing by 12 weeks gestation, but the consumption of prenatal iron-folate was negligible in the Pakistan site. Women who had twin pregnancies were not excluded.

The Data Coordinating Center (DCC) created the randomization scheme and centrally generated the allocation sequence for each site. The scheme included a permuted block design stratified by GN clusters for assigning individual participants to a trial arm. The allocation ratio was 1:1:1 within blocks which randomly varied between sizes of 3, 6, or 9 for each site. Once an eligible participant was identified, the randomization assignment was generated by the site data manager from the centralized computerized data management system maintained by the DCC.

### Nutrition intervention

The principal intervention was a lipid-based micronutrient supplement (Nutriset, Melauney, France) which also provided a favorable balance of polyunsaturated fatty acids and small quantities of protein and energy [32]. This product had minor modifications to a product previously designed for pregnant/lactating women [33]. Participants were instructed to take one sachet daily until delivery.

An additional protein-energy supplement was provided to women whose BMI was <20 kg/m^2^ from the time of randomization for Arm 1 participants and from the beginning of the 2^nd^ trimester for Arm 2 participants. This protein-energy supplement was also provided to any Arm 1 or Arm 2 participant who failed to meet guidelines for gestational weight gain in the 2^nd^ and 3^rd^ trimesters [34]. Once the second supplement was commenced for either reason, it was provided until delivery. More than 90% of the women who had live births with newborn outcome measures received this additional protein energy supplement during the second and third trimesters.

### Anthropometry and measurement of fetal crown-rump length (CRL)

Neonatal recumbent lengths, weights, and head circumferences were obtained within 48 hours of delivery by trained and periodically certified assessment teams. Measurements were obtained in triplicate, entered into the database, and the median value used for analysis. Equipment used were neonatal stadiometers (Ellard Instrumentation Ltd, Monroe WA), seca 334 electronic scales, and seca 201 measuring tapes (seca North America, Chino CA). CRL measurements were obtained in the first trimester by ultrasonography.

### Home visits and compliance

Participants in all three arms were visited by the home visitor research assistants every two weeks to record an interim health history and to administer a urine pregnancy test. The pregnancy testing was combined with calendar records of menses to ascertain last menstrual period and to guide the timing of ultrasounds to be obtained between 10–12 weeks estimated gestation. For Arms 1 and 2, these visits were also used to replenish the supply of trial supplements. Compliance with supplement(s) use was documented by inspection of calendars the women completed daily and by collection of empty, partially eaten, and unused intervention sachets. Compliance was calculated for Supplement 1 as the total number of sachets fully eaten divided by the number of days between starting Supplement 1 and delivery. Supplement 2 compliance was calculated similarly; however, the numerator is the total number of Supplement 2 sachets fully or partially eaten.

### Adverse events and safety monitoring

Adverse events were monitored continuously as per protocol [32] and reported to the overall study principal investigators and the DCC within 48 hours for all deaths and within seven days for other adverse events, including adverse pregnancy outcomes, adverse neonatal events, hospitalizations, and allergic reactions. The Data Monitoring Committee reviewed the study progress for safety, trial progress, data completion, supplement compliance, and protocol violations twice yearly.

### Statistical methods

The Intergrowth-21^st^ standards were applied to describe the results of newborn anthropometry [35, 36] in terms of Z-scores and centiles (not shown except for weight-for-age centile <10th percentile). Study outcomes were assessed with a modified intention-to-treat approach. The overall treatment effect and pairwise comparisons (Arm 1 vs. Arm 2, Arm 1 vs. 3, as well as Arm 2 vs. 3) for the continuous anthropometry outcomes were obtained from linear models for the continuous outcomes (newborn length, weight, HC, LAZ, WAZ, HCAZ, and WLRAZ). Model-generated measures of ES with 95% confidence intervals (CI) and P values were adjusted for site and GN cluster nested within site. For binary anthropometry outcomes (SGA, LBW, PTB, LAZ <-1, LAZ <-2, WAZ <-1, WAZ <-2, WLZ <-1, WLZ <-2, WLRAZ <-1 and WLRAZ <-2), generalized linear models with generalized estimating equations were utilized to calculate relative risks with 95% CI and P values after adjusting for site while controlling for GN cluster correlations. P values are presented for descriptive purposes.

## Results

### Maternal characteristics

**Fig 1:**
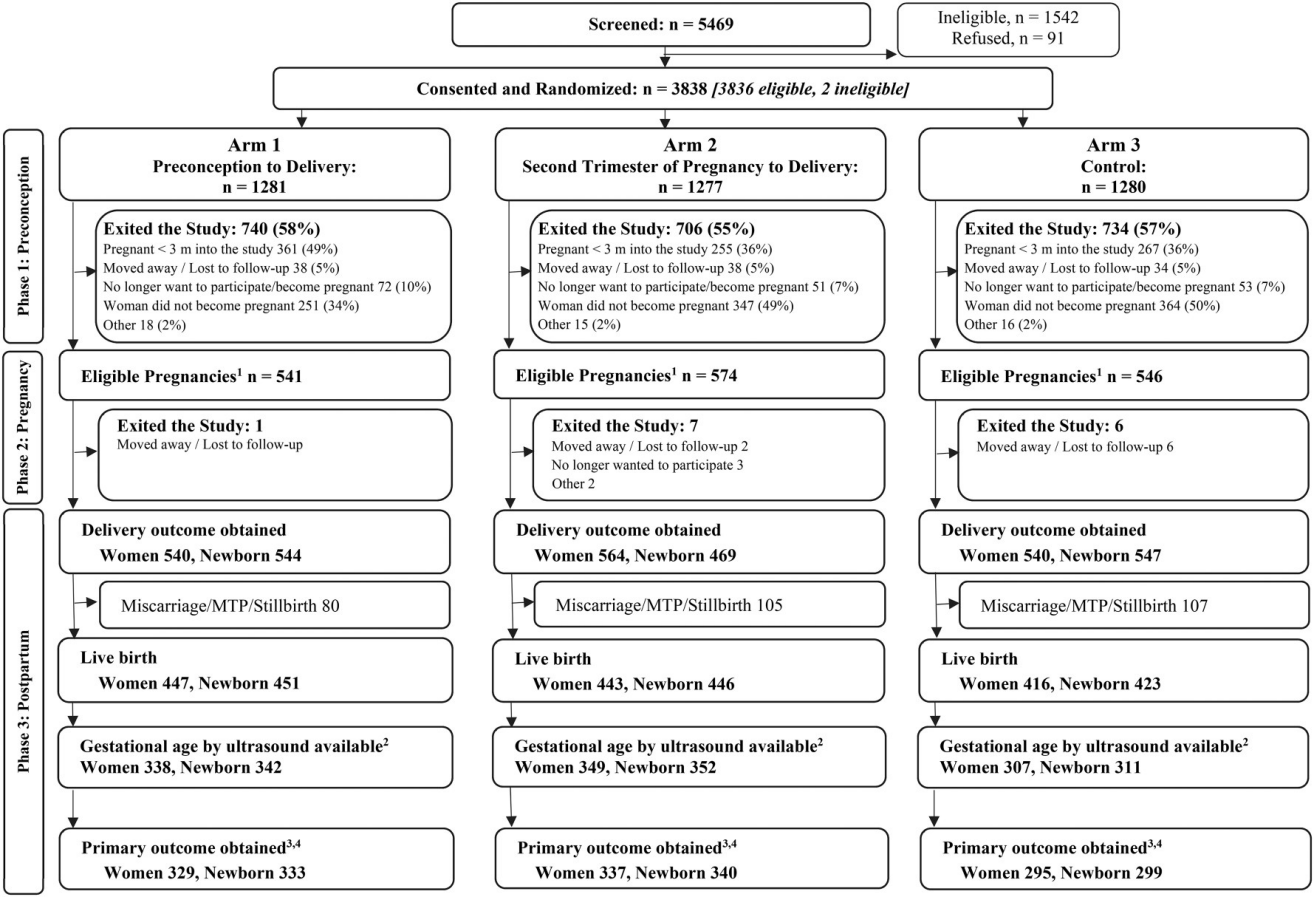
Consort Diagram for Women First Preconception Maternal Nutrition Trial in South Asian region. ^1^Excludes women who became pregnant <3 months into the study. The women who had eligible pregnancies may have had delivery data obtained or they may have exited the study prior to delivery. ^2^Gestational age (GA) at birth is defined as the age at the time of the ultrasound based on the ultrasound plus time until birth if the ultrasound was done between 6 weeks + 0 days to 13 weeks + 6 days and the GA at birth is between 24 weeks + 0 days and 42 weeks + 6 days. ^3^Primary outcome was obtained for live newborns with three length measurements taken within 48 hours of delivery. ^4^Length-for-age, weight-for-age, head circumference-for-age, and weight-length-ratio-for age Z-scores and percentiles based on measurements within 48 hours of age are calculated using the INTERGROWTH-21st International Standards for Newborn Size [37] and International Standards for Newborn Size for Very Preterm Infants [38] which provide Z-scores by sex and GA at birth for infants born between 33 weeks + 0 days to 42 weeks + 6 days GA at birth and between 24 weeks + 0 days to 32 weeks + 6 days GA at birth, respectively.

Women First participants in the combined S. Asian sites who were screened, enrolled and randomized, had eligible pregnancies, first trimester ultrasound determination of gestational age, delivered live births, and whose newborn had measurements within 48 hours are given in Fig 1. Nine hundred and sixty one pregnancies (972 newborns) for the combined S. Asian sites qualified for inclusion in this analysis. The number of pregnancies ranged from 295–337 per arm (Table 1). Baseline maternal characteristics for S. Asian mothers with a primary outcome differed between arms only for maternal education, with a higher percentage of women in Arms 1 and 3 having no formal education (p = 0.04, Table 1). Mean ± SD duration of exposure to Supplement for Arm 1 was 72.6 ± 16.8 weeks with overall compliance of 89.6 ± 9.9%. For Arm 2, duration of exposure to Supplement 1 was 26.3 ± 2.2 weeks with estimated compliance of 87.7 ± 15.7%. Supplement 2 was started in 67% of Arm 1 women prior to conception and in an additional 29% during gestation. In Arm 2, 92% of the women received Supplement 2 starting after 12 weeks gestation. The duration of Supplement 2 was 56.2 ± 25.5 weeks for Arm 1 and 23.7 ± 5.1 weeks for Arm 2.

**Table 1:** Overall baseline characteristics among women in South Asian study sites who had the primary outcome and gestational age available for newborns by treatment arm^a^.

Variable	Arm 1 (n = 329)	Arm 2 (n = 337)	Arm 3 (n = 295)
Maternal age, n (%)			
<20	79 (24.0)	80 (23.7)	61 (20.7)
20–24	148 (45.0)	146 (43.3)	124 (42.0)
25 +	102 (31.0)	111 (32.9)	110 (37.3)
Maternal education, n (%)			
No formal schooling	152 (46.2)	137 (40.7)	133 (45.1)
Primary	28 (8.5)	48 (14.2)	46 (15.6)
Secondary +	149 (45.3)	152 (45.1)	116 (39.3)
Body Mass Index (BMI), kg/m^2^			
Mean ± SD	19.9 ± 3.1	19.7 ± 3.0	19.7 ± 3.1
Median (P25—P75)	19.4 (17.8, 21.7)	19.4 (17.6, 21.4)	19.3 (17.4, 21.5)
BMI < 20.0, n (%)	181 (55.0)	190 (56.4)	174 (59.0)
BMI < 18.5, n (%)	122 (37.1)	127 (37.7)	113 (38.3)
Height, cm, n			
Mean ± SD	152.0 ± 5.9	151.2 ± 6.5	151.9 ± 6.2
Median (P25—P75)	151.9 (148.5, 156.0)	150.8 (147.0, 155.1)	151.5 (148.3, 156.0)
Parity, n (%)			
0 (nulliparous)	110 (33.4)	85 (25.2)	76 (25.8)
1	111 (33.7)	118 (35.0)	95 (32.2)
≥ 2	108 (32.8)	134 (39.8)	124 (42.0)
Tally of indicators of higher SES^b^, n (%)			
None (0 present)	6 (1.8)	2 (0.6)	6 (2.0)
1–2 present	92 (28.0)	87 (25.8)	68 (23.1)
3–4 present	159 (48.3)	183 (54.3)	160 (54.2)
5–6 present	72 (21.9)	65 (19.3)	61 (20.7)

^a^Primary outcome obtained from one or more newborn of the woman.

^b^The socio-economic status (SES) tally provides the number of indicators available from the following list: electricity, improved water source, sanitation, man-made flooring, improved cooking fuels, and household assets.

### Newborn outcomes

The number of newborn with anthropometric outcomes ranged from 299–340 per arm (Table 2). Fortuitously the twin pregnancies were equally distributed between the three arms accounting for 2.5% of total newborn included in this analysis.

**Table 2:** Women First Maternal Preconception Nutrition Trial in South Asia. Growth outcomes by treatment arm among all livebirths with gestational age at birth^a^ available and length measurements within 48 hours after birth.

	Treatment Group		
Variable	Arm 1 (n = 333)	Arm 2 (n = 340)	Arm 3 (n = 299)
Length (cm)			
Mean ± SD	47.71 ± 2.21	47.60 ± 2.59	47.18 ± 2.60
Median (P25, P75)	47.90 (46.50, 49.00)	47.80 (46.30, 49.20)	47.40 (45.80, 48.80)
Length-for-Age Z-Score (LAZ)^b^			
Mean ± SD	-0.62 ± 0.99	-0.69 ± 1.13	-0.90 ± 1.15
Median (P25, P75)	-0.55 (-1.24, -0.03)	-0.68 (-1.44, 0.07)	-0.88 (-1.74, -0.12)
LAZ <-1, n (%)	110 (33.0)	130 (38.2)	134 (44.8)
LAZ <-2, n (%)	33 (9.9)	45 (13.2)	53 (17.7)
Weight (gm)			
Mean ± SD	2757 ± 451	2714 ± 459	2668 ± 431
Median (P25, P75)	2785 (2480, 3030)	2730 (2455, 3003)	2660 (2400, 2955)
Weight-for-Age Z-Score (WAZ)^b^			
Mean ± SD	-0.98 ± 0.97	-1.11 ± 0.97	-1.20 ± 0.99
Median (P25, P75)	-0.98 (-1.63, -0.36)	-1.16 (-1.73, -0.43)	-1.25 (-1.97, -0.53)
WAZ <-1, n (%)	164 (49.2)	198 (58.2)	180 (60.2)
WAZ <-2, n (%)	52 (15.6)	55 (16.2)	68 (22.7)
Weight-Length Ratio-for-Age Z-Score (WLRAZ)^b^^,^^c^			
Mean ± SD	-1.38 ± 1.37	-1.58 ± 1.32	-1.65 ± 1.39
Median (P25, P75)	-1.35 (-2.30, -0.55)	-1.71 (-2.38, -0.65)	-1.70 (-2.67, -0.72)
WLRAZ <-1, n (%)	200 (61.0)	233 (69.1)	202 (68.5)
WLRAZ <-2, n (%)	105 (32.0)	126 (37.4)	125 (42.4)
Head circumference (HC, cm)^d^			
Mean ± SD	32.94 ± 1.43	32.87 ± 1.55	32.78 ± 1.60
Median (P25, P75)	33.00 (32.00, 33.90)	33.00 (32.10, 33.80)	32.90 (32.00, 33.80)
HC-for-Age Z-Score (HCAZ)^b^^,^^d^			
Mean ± SD	-0.55 ± 1.05	-0.62 ± 1.06	-0.66 ± 1.15
Median (P25, P75)	-0.60 (-1.27, 0.15)	-0.59 (-1.31, 0.01)	-0.70 (-1.44, -0.02)
HCAZ < -1, n (%)	116 (34.9)	119 (35.1)	110 (36.8)
HCAZ < -2, n (%)	27 (8.1)	29 (8.6)	32 (10.7)
Small for gestational age, n (%)^e^	121 (36.3)	148 (43.5)	147 (49.2)
Low birth weight (<2500 gm), n (%)	92 (27.6)	98 (28.8)	102 (34.1)
Preterm birth (<37 weeks GA), n (%)	41 (12.3)	29 (8.5)	37 (12.4)

^a^Gestational age (GA) at birth is defined as the gestational age at the time of ultrasound measurement of fetal crown-rump length plus time until birth if the ultrasound was done between 6 weeks + 0 days to 13 weeks + 6 days and the GA at birth is between 24 weeks + 0 days and 42 weeks + 6 days.

^b^Length, weight, head circumference, and weight-length-ratio z-scores and percentiles based on measurements within 48 hours of age are calculated using the International Standards for Newborn Size and International Standards for Newborn Size for Very Preterm Infants published by the INTERGROWTH-21^st^ Project [37–39] which provide Z-scores and percentiles by sex and GA at birth for infants born between 33 weeks + 0 days to 42 weeks + 6 days GA at birth and between 24 weeks + 0 days to 32 weeks + 6 days GA at birth respectively.

^c^Arm 1 n = 328; Arm 2 n = 337; Arm 3 n = 295; total n = 960.

^d^Arm 1 n = 332; Arm 2 n = 339; Arm 3 n = 299; total n = 970.

^e^Small for Gestational Age (SGA) is a classification given to infants with a low birth weight, more specifically, a birth weight that is in 10^th^ percentile or lower based on standards by gestational age at birth and sex developed by INTERGROWTH-21^st^ Project.

Anthropometric measures for controls (Arm 3) were determined by sex. Mean ± SD lengths were 46.83 ± 2.48 and 47.53 ± 2.67 cm for females and males, respectively. Corresponding figures for weight were 2605 ± 421 and 2731 ± 433 g and for head circumference were 32.44 ± 1.62 and 33.12 ±1.51 cm. However, the effects of the maternal supplements on newborn anthropometry did not vary by sex (p = 0.2133). Accordingly, sexes have been combined for all analyses.

For the newborns of women who did not receive trial nutrition supplements (Arm 3), mean LAZ and WAZ were low with WAZ more impaired than LAZ. This disparity was reflected in the WLRAZ with a mean of -1.65, but considerable variability (standard deviation 1.39) (Table 2). Head circumference was least impaired with a mean HCAZ of -0.66. Eighteen percent of newborns in Arm 3 were stunted (LAZ <-2) and 42% were wasted (WLRAZ <-2). For more than two-thirds of the newborns, the WLRAZ was <-1. Twelve percent were preterm deliveries; thirty-four percent had LBW, and 49% of Arm 3 were SGA. Head circumference was <-2 Z-scores in 11% of Arm 3.

**Fig 2:**
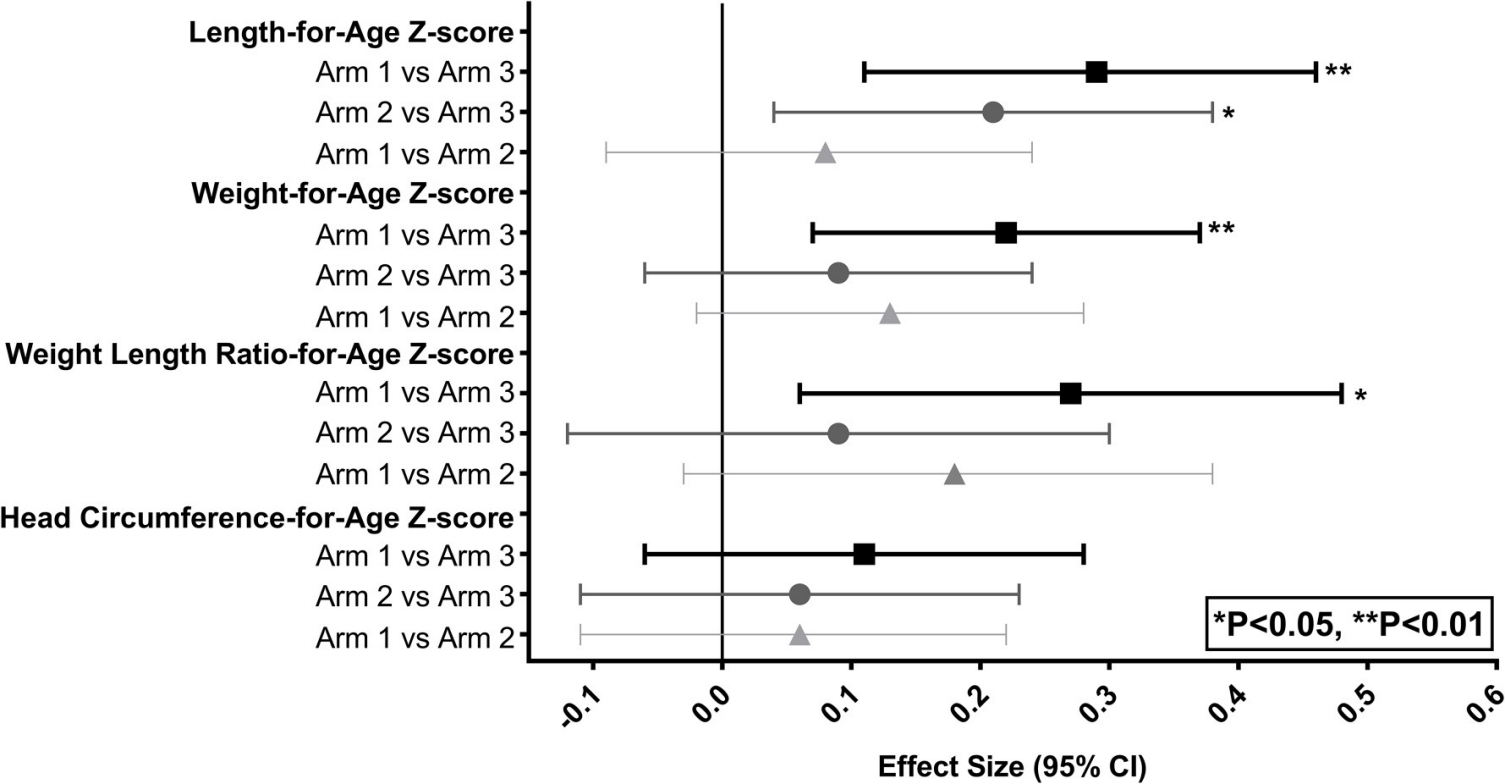
Women First Preconception Maternal Nutrition Trial: Effect sizes (95% confidence intervals) for continuous newborn continuous anthropometric outcomes in South Asian region.

Ninety-five percent CI for the positive effect sizes of the nutrition intervention commencing ≥ 3 months prior to conception (Arm 1) in comparison with control Arm 3 did not include zero for LAZ, WAZ, and WLRAZ. In contrast, corresponding CI for effect sizes for continuous variables for the nutrition intervention commencing in the first trimester of pregnancy (Arm 2 vs. 3) included zero with the only exception of LAZ (Fig 2).

**Fig 3:**
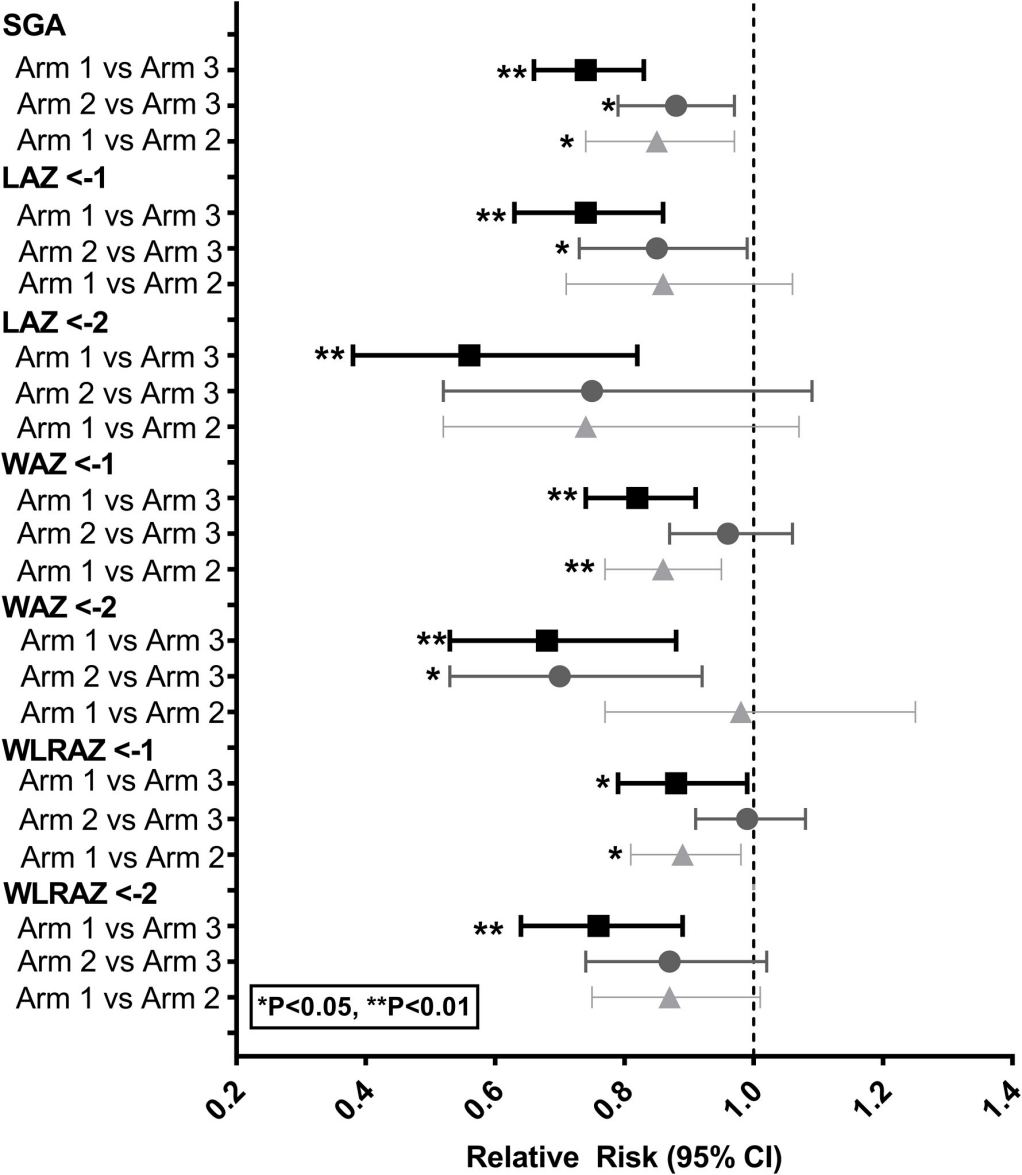
Women First Preconception Maternal Nutrition Trial: Relative risks (95% confidence intervals) for binary newborn anthropometric outcomes in South Asian region. LAZ, length-for-age Z-score; SGA, small-for-gestational age; WAZ, weight-for-age Z-score; WLRAZ, weight to length ratio-for-age Z-score.

For binary variables, there was no reduction in relative risk for preterm delivery for either the preconception (Arm 1) or 1^st^ trimester intervention (Arm 2) compared to the control arm (Arm 3). The intervention commencing in the preconception period (Arm 1) resulted in a reduction of 26% in the relative risk of SGA (p<0.0001) and a reduction in the incidence of LBW of 19% ((RR 0.81 (0.66–1.00, p = 0.0461)) in comparison with control arm (Arm 3). There were also substantial reductions (p<0.01) in the relative risks for stunting (LAZ <-2, 44%), underweight (WAZ <-2, 32%), and wasting (WLRAZ <-2, 24%) (Fig 3). For the intervention commencing in the first trimester (Arm 2) compared with the control arm (Arm 3), several outcomes were marginally different (P<0.05) but none of these outcomes differed at α = 0.01 level. Direct comparison of Arm 1 vs Arm 2 showed reduced risk for SGA, WAZ<-1 and WLRAZ<-1 for the preconception intervention. The only suggestion of the intervention commencing at either time in relation to conception having a benefit on head size was a RR for head circumference <3^rd^ centile for Arm 1 vs. 3 of 0.73 (95% CI: 0.53–1.00).

## Discussion

These newborn anthropometric outcomes for the combined data from the two S. Asian sites included in the Women First trial demonstrate that substantial corrections of deficits in both weight and linear measures of fetal growth can be achieved with maternal nutrition supplements commenced in the first trimester or earlier. Despite lack of significant differences for direct comparisons between Arms 1 and 2, the point estimates for the effect sizes were consistently more favorable for continuous and relative risks for binary anthropometric outcomes for the preconception intervention. These advantages of improving the nutrient and energy intake of women of childbearing age prior to conception were documented in S. Asian trial populations unselected for past or current nutritional status. These rural populations were, however, characterized by low income and sub-group dietary data in the first trimester documented low intakes of multiple micronutrients and, especially in India, borderline protein-energy intake [40].

The goal of this trial was not to evaluate a specific nutrition product or combination of products but rather to use these products to advance our understanding of the extent to which deficits in fetal growth could be corrected by commencing nutrition interventions very early in pregnancy or for a substantial time prior to conception in women of childbearing age in these and similar populations. It is quite possible that greater effects could be achieved with further modifications of the interventions, perhaps especially if guidelines for maternal gestational weight gain could be matched, and that these may be achievable with sustainable solutions including education and dietary improvements [41]. Meanwhile, the results of this trial extend our knowledge of the extent to which fetal growth deficits in poor S. Asian populations are attributable to undernutrition alone and can be corrected with nutrition interventions in women of childbearing age.

This trial addressed persistent shared and prominent challenges in this region [1]. A trial of iron-folate supplementation (IFS) early in this century documented a modest increase in birth weight, reduction in risk of low birth weight, and increase in head circumference [42–44]. The additional benefits of maternal multiple micronutrient supplements beyond those receiving IFS alone, either as trial supplements or standard of care, have been reviewed in detail [45], including data from India, Pakistan, Nepal, and Bangladesh. This analysis confirmed significantly but quite modestly lower relative risks for LBW, PTB, and SGA. A major contributor to this meta-analysis was the JiViTA-3 trial in Bangladesh, in which significant reductions in LBW were attributable to a longer mean gestational age and lower incidence of PTB rather than any effect on fetal growth rates [46]. This finding differed from the Women First trial in which gestational ages at delivery were the same for the three trial arms. The prematurity incidence in the control arm for the S. Asian sites in the current study (12.4%) was similar to that recently estimated for S. Asia [47]. The results for the meta-analysis of Smith, et al., on birth size were similar to those of an earlier meta-analysis of data from 12 sites [48] including four in S. Asia, one each in Pakistan [49] and Bangladesh [50], and two in Nepal [42, 51]. There has been only one lipid nutrition supplement trial in S. Asia that has been reported prior to Women First [52]. In comparison with the IFS control arm, this trial in rural Bangladesh resulted in statistically significant improvements in mean birth weight and length and in binary outcomes associated with these measurements. However, with the exception of sub-group analyses and reduction in the rate of stunting, all improvements were very modest.

The effect sizes for continuous outcomes and relative risks for binary outcomes in the current study compared favorably with those of previous trials in S. Asian countries in terms of improvements in fetal growth deficits whether the interventions were iron folate supplements, multi-micronutrients, or lipid nutrition supplements. The extent of the contribution to favorable outcomes of the additional protein-energy supplements is unknown. These were provided to two-thirds of Arm 1 participants prior to conception and to more than 90% of both Arm 1 and 2 participants during the 2^nd^ and 3^rd^ trimesters. Significant increases in birth weight resulting from maternal balanced protein energy supplements during pregnancy have been documented in S. Asia [53] and more widely [54]. However, this has not been a consistent finding [55]. Perhaps relevant and in contrast to the results of the Women First trial [30], these increases in birth weight in previous trials were specifically not accompanied by any increases in birth length. Though we did not observe any statistical differences between Arms 1 and 2 for newborn LAZ, the preconception (Arm 1) anthropometric outcomes had the largest point estimates for effect size compared to Arm 3 in this trial and compared favorably with corresponding outcomes of previous studies commenced during gestation. Both separately and combined with the data for the first trimester arm, these results are strongly supportive of commencing nutrition interventions as early as possible for women of childbearing age in resource poor populations.

A limitation of this trial has been its inevitable restriction to only part of one maternal life and to targeting only one environmental factor, namely maternal nutrition, when challenged with a multifactorial etiology [56]. The strengths of this trial include an intervention relatively comprehensive in nutrient content and quantity. The principal strength has been the early start of the intervention, especially the preconception arm, and the inclusion of women of childbearing age in these resource-poor communities irrespective of their long- or short-term nutritional status as defined by anthropometric indices.

## Conclusions

Newborn anthropometric data for the Women First trial in the S. Asian region (combined data for the sites in India and Pakistan) provide further confirmation of the high incidence of stunting, wasting, low birth weight, and SGA in this region. Commencing a relatively comprehensive nutrition supplement at least three months prior to conception for women unselected for anthropometric indicators of nutritional status was associated with decreases of 44% in stunting, 24% in wasting, and 26% SGA when compared to the control group. These results provide quantitative information on the benefits to fetal growth achievable by improvement in intake of energy, protein, and micronutrients commencing before the start of the second trimester of gestation without any other interventions directed to improvement of the poor environments in which the participants lived. Furthermore, the relatively large point estimates for the effects of the nutrition supplement commenced ≥ 3 months prior to conception support enhanced efforts to improve the nutrition of all women of child-bearing age in resource-poor populations in these and similar environments in order to diminish impairment of fetal growth.
